# Evaluation of the Neurological Complaints during Pregnancy and Postpartum

**DOI:** 10.31661/gmj.v8i0.1616

**Published:** 2019-12-31

**Authors:** Shaghayegh Zafarmand, Haniyeh Javanmardi, Maryam Ameri, Masoud Maneshi, Susan Mansouri-Mehrabadi, Yasaman Zolghadrasli, Mahshad Moazzam, Ayda Aramesh, Afshin Borhani-Haghighi

**Affiliations:** ^1^Clinical Neurology Research Center, Shiraz University of Medical Sciences, Shiraz, Iran; ^2^Student Research Committee, Shiraz University of Medical Sciences, Shiraz, Iran; ^3^Department of Neurology, School of Medicine, Shiraz University of Medical Sciences, Shiraz, Iran

**Keywords:** Neurological Disorders, Pregnancy, Postpartum

## Abstract

**Background:**

Pregnancy and postpartum are critical periods for patients with neurological complications. In this study, we aimed to evaluate the clinical characteristics and outcome of pregnant women with neurological conditions.

**Materials and Methods:**

This cross-sectional study reviewed pregnant women with neurological signs and symptoms, who were registered in the Medical Care Monitoring Center (MCMC) database of Shiraz University of Medical Sciences 2013-15. A questionnaire was designed to record each patient’s information including demographic variables, past medical history, clinical presentation, obstetric profile, and fetal/maternal outcomes.

**Results:**

Totally, 332 mothers were registered in the database. The main neurological complaints in our population were headache, seizure, unilateral neurological symptoms, multiple sclerosis, neuromuscular disorder, and brain tumor. More than half of the patients (54%) experienced headache during the pregnancy and postpartum period.

**Conclusion:**

Evaluating the neurological disorders separately, based on the time of symptom onset indicates the importance of follow-up of mothers during peripartum. Our findings suggest that decisions for pregnancy in women with neurological disorders should be based on risks outweighing for the mother and the fetus, particularly regarding the pharmacological side effects.

## Introduction

Pregnancy and postpartum are critical periods in developing neurologic complications. These periods are associated with significant physiological and hormonal changes in women, which might cause relapse or remission of previous neurological disorders or cause a new one [1, 2]; appropriate diagnosis and management of these conditions are vital in ensuring maternal and fetal safety. Some of the common neurologic disorders that pregnant women encounter include headache, seizure, cerebrovascular accidents, eclampsia /preeclampsia, and mononeuropathies [3, 4]. We designed a cross-sectional study to assess the prevalence and outcome of each neurological symptoms or disease during pregnancy and peripartum period amongst high-risk mothers registered in the Medical Care Monitoring Center (MCMC) affiliated with Shiraz University of Medical Sciences, Shiraz, Iran.

## Materials and Methods

This cross-sectional study was performed among high-risk mothers with neurological signs or symptoms while pregnant or six weeks postpartum. We collected the data from the MCMC registry system, which was launched in 2012 to record and promote the health of at-risk pregnant women. A pregnancy in which the risk of maternal and fetal mortality and disability is more than normal, considered as a high-risk pregnancy. In this system, demographic and clinical features of all pregnant women with positive free-beta-human chorionic gonadotropin (free-beta-hCG) test and a fetal/maternal life-threatening disease that required hospital admission (previous or newly diagnosed disorders) was recorded. We designed a questionnaire including set of demographic variables, obstetric profile (parity, gravidity, gestational diabetes, gestational hypertension), past medical history, clinical characteristics (type of neurologic symptoms, time of onset, duration), as well as fetal and maternal outcomes (delivery method, premature delivery, fetal disorders and death, modifying ranking scale [MRS] of mother). In addition, patients were followed-up for 6 weeks after delivery, using a phone call or in-person interviews in case if they were accessible. Before commencing the study, verbal consent was obtained from all participants. Variables were analyzed using statistical package for Social Sciences, version 16.0 (SPSS Inc., Chicago, Ill., USA) and presented as counts and percentages.

## Results

A total of 705 at-risk pregnant women had registered in MCMC from April 2013 till November 2015, out of which, 332 patients had the inclusion criteria. The mean age of patients was 29.32 years old. Table-1 describes the patients’ characteristics in this study. With respect to obstetric profile and pregnancy outcome, 19% of them had nonproteinuric gestational hypertension and 9% had gestational diabetes. Most mothers and their newborns were in good condition during six weeks follow-up without any complications. In total, two mothers died during delivery and the incidence of stillbirth, fetal abortion, preterm delivery, and congenital anomalies were 2%, 5%, 8%, and 0.6%, respectively. Other obstetric and neurological profiles and pregnancy outcome are also summarized in Table-1. Table-2 shows the characteristics, neurological profile and pregnancy outcome of patients with each neurological condition, separately. Table-3 shows the onset time of each neurological symptoms. The prevalence of each neurological disorder is reported below.

### Headache

More than half of the patients (54%) experienced headaches during their pregnancy and postpartum period. Sixty-six percent developed primary headaches (mainly migraine and tension headaches). The remaining headaches were secondary to infection and preeclampsia (34%). Twenty percent of all headaches occurred during the first trimester, 20% in the second and 28% in the third trimester. Also, 1% occurred at the time of giving birth or after birth, and finally 31% during their postpartum period. Gestational hypertension and gestational diabetes were the most common pregnancy complications in patients with headaches, which was observed in 19.5% and 10%, respectively. Regarding maternal and fetal outcomes, 80% of infants were born healthy at term gestation, while 20% were aborted. Also, 83% of the pregnant women with headaches gave birth at term gestation, out of which, 65% were delivered by cesarean section (C/S) and the reaming (35%) were born via normal vaginal delivery (NVD). Three percent of mothers experienced decreased levels of consciousness or coma. The prevalence of preterm labor in mothers with headache was 8%. Amongst these mothers, stillbirth and congenital anomalies were observed in 0.6% of newborns.

### Seizure

The seizure was observed in 17.2 % of pregnant women, and 67% of patients with seizures had the previous history of epilepsy. Regarding seizure occurrence time, 27% of women experienced seizure in their postpartum period. The rate of seizure in the first, second, and third trimester was 3%, 19%, and 22%, respectively. Eclampsia accounted for 16% of these seizures, 9% was drug-induced, and 4% occurred without any specific cause. Hypertensive disorder of pregnancy and gestational diabetes were observed in 25% and 9% of patients with seizure. About 73% of neonates were born healthy at term. The rate of NVD and C/S was 37% and 63%. Abortion, preterm birth, and stillbirth occurred in 5.5%, 2%, and 11% of our study population.

### Unilateral Neurological Symptoms

About 12% of pregnant women in our study were hospitalized due to unilateral neurological symptoms, of which, 73% had cerebral venous sinus thrombosis (CVST), 7.5% had ischemic stroke and 20% had cerebral hemorrhage (intracranial or subarachnoid hemorrhage). Gestational hypertensive disorders and diabetes were observed in 15% and 7.5% of the patients. About 13% of pregnant women with unilateral neurological symptoms were presented with a decreased level of consciousness or coma. About 73% of neonates were born healthy at term. Twenty-one percent of neonates were born by NVD and 79% via C/S. Abortion, preterm delivery, and stillbirth occurred in 2.5%, 5%, and 7.5% of our patients, respectively. The rate of these symptoms in the first, second, and third trimester was 8%, 19.5%, and 26%, respectively. About 5% of these symptoms occurred during delivery and 50% in postpartum.

### Multiple Sclerosis (MS)

About 4.5% of pregnant women in this study had MS. Near 13% of them were new cases and all were diagnosed in the first trimester of pregnancy. Gestational diabetes was observed in 7% of these patients, but none had gestational hypertension. The rate of abortion was 20% and no stillbirth, preterm delivery or congenital anomalies were observed amongst our patients. Eighty percent of the infants were born at term, out of which, 67% were born via C/S and 33% by NVD.

### Neuromuscular Disorders

About 4.5% of our patients suffered from peripheral neuromuscular diseases. Four patients had myasthenia gravis (MG), and one had Guillen-barre syndrome (GBS). Neither Carpal tunnel syndrome (CTS) nor Bell’s palsy was observed in our population. About half of these patients (50%) reported having a positive history of neuromusculardisorders before pregnancy and the remaining developed neuromusculardisorders symptoms in the second trimester of their pregnancy for the first time. Symptoms of the newly diagnosed patients were exacerbated during six months follow-up. All the infants were born healthy at term. Four neonates were born *via* C/S and one by NVD. No neonatal and maternal complications were observed in this group.

### Brain Tumors

Two cases in our population had brain tumor, and one had gestational diabetes. One infant was delivered at term while the other was delivered preterm, and both were delivered via C/S. The onset of symptoms was in the first and the second trimesters for both patients.

## Discussion

In this study, major neurological complaints were classified as headache, seizure, unilateral neurological symptoms, multiple sclerosis, neuromuscular disorder, and brain tumor. Amongst mothers, the headache was the most common neurologic symptom, referring to Shiraz health centers. Primary headache was observed in two-third of mothers, that were alleviated during pregnancy with minimum intervention. Secondary headaches occurred in one-third of patients while infection and preeclampsia were the main causes. Typically, primary headaches diminish during pregnancy, but in many cases, headaches begin or worsen in this period [4]. As previous studies claimed, migraine is the most sensitive primary headache to the ovarian hormonal changes; hence, it is a common complication during pregnancy [4, 5]. Similarly, in our study, migraine was the most prevalent complaint. Managing these headaches during pregnancy and postpartum is similar to its management in none pregnant women, with a few exceptions [5]. Secondary headaches might begin during pregnancy, and can be due to vasculitis, idiopathic intracranial hypertension, pituitary and brain tumor, arteriovenous malformation, CVST, stroke, subarachnoid hemorrhage, pre-eclampsia and eclampsia [5-7]. In this study, the most common underlying causes of secondary headaches were infections and preeclampsia. A high prevalence of headaches in the postpartum period, as observed in this study, might be associated with dramatic decline of estrogen level after pregnancy [8]. In our study, gestational hypertension and gestational diabetes were the most common pregnancy complications in patients with headache. Also, previous studies revealed that headaches, especially migraines, highly increased the risk of hypertensive disorders during pregnancy [9]. In this study, 83% of infants of pregnant women with headaches were born at term. Similarly, several studies on the outcome of pregnancy in women with headache showed that there was no significant difference between mothers with and without headaches with respect to the outcome [10, 11]. Epilepsy and seizure disorders are the most frequent neurological conditions in obstetrics after migraine [12]. The majority of patients with seizure during pregnancy had a positive history of seizure attacks before pregnancy [13, 14]. Previous epidemiological studies estimated that between 0.3-0.5 of all births were attributed to pregnant women with seizure [12]. In our study, about 17.2 % of pregnant women with neurological deficits were presented with seizure. There are many physiological changes during pregnancy that can affect seizure activity in epileptic women. Decreased albumin level, increased renal blood flow, and increased hepatic metabolism and CYP-450 activity might contribute to increased or altered drug availability, lead to seizure attack in pregnant women [15]. The majority of patients with seizure during pregnancy have a positive history of seizure attack before pregnancy [13, 14]. Epilepsy, eclampsia, cerebral venous sinus thrombosis, drug reactions, and ICH were the most common causes of seizure during pregnancy [13, 14]. In our study, eclampsia accounted for 16% of all seizures, 9% was drug-induced, and 4% occurred without any specific reason. Many studies have claimed that the risk of pregnancy complications such as hypertension, eclampsia/preeclampsia, and cesarean delivery as well as neonatal complications were higher in women with seizure in comparison with the general population [16-19]. On the contrary, others showed no significant correlation between seizure and maternal or fetal complications [20-22]. In our study, hypertensive disorders during pregnancy were observed in a quarter of patients with seizure. Coma and decreased consciousness level were not rare amongst mothers with convulsion. Moreover, the rate of C/S, abortion, preterm birth, and stillbirth increased in mothers with seizure. As observed in this study, most women experienced seizure in their postpartum period and the rate of seizure increased by the gestational age. These findings suggest that following up on patients during the postpartum period for seizure attack is highly necessary. Also, applying any seizure prophylaxis seems to be crucial to develop the neonatal and obstetric outcomes in patients with positive history of seizure attack, especially during the third trimester of pregnancy and postpartum period. The majority of our patients with unilateral neurological symptoms had CVST, and the remaining had ischemic stroke or cerebral hemorrhage. The higher risk of CVST and ischemic stroke could be contributed to the hypercoagulable state of pregnancy, resulting from increase in the production of coagulation factors (factor VIII, von Willebrand, fibrinogen), decrease in protein S level, protein C resistance, and downregulation of fibrinolysis [23]. Furthermore, hormonal and hemodynamic changes during pregnancy affect the vessels wall; hence, the risk of the aneurysm and cerebrovascular accident increases during pregnancy [24]. Also, high blood pressure, hemolysis, and elevated liver enzymes in pregnancy might lead to ischemic or hemorrhagic stroke [23]. In line with our study, previous studies showed that unilateral neurological symptoms were more common in the third trimester and postpartum period [24]. In this study, about half of the unilateral neurological symptoms occurred during the post-partum period. Estrogen and progesterone are immunomodulatory hormones, which increase during pregnancy, lasting until delivery and then returning to baseline level during postpartum. Consequently, pregnancy appears to have a protective effect on MS disease with fewer and less severe relapses, especially in the third trimester due to high estrogen and progesterone level. Studies show that the exacerbation rate of MS increases in the first 3 months after delivery. In this study, all patients with MS were diagnosed in the first trimester and there was no MS symptom in the second and third trimester. Several studies showed no signiﬁcant increase in obstetric and neonatal complications for patients with MS in comparison with the normal controls [25]. However, we observed abortion in 20% of mothers with MS. Therefore, these mothers should be advised regarding the risk of abortion. CTS and Bell’s palsy are the most common neuromuscular diseases in pregnancy, occurring due to fluid retention and soft tissue edema in this period [26]; however, no CTS and Bell’s palsy were observed in our population. GBS and MG are both autoimmune disorders that theoretically should improve during pregnancy due to the immunomodulatory effect of estrogen and progesterone [27]. But in this study, MG and GBS were the most common neuromuscular disorders. Similarly, in a study amongst pregnant women with MG, the disease exacerbated during pregnancy in one-third of cases while in two-third of them symptoms did not change [27].In patients diagnosed with GBS in the second trimester of pregnancy, the symptoms exacerbated during six months follow-up. In a study by Wiertlewski *et al*. (2004) and in a study by Brook *et al*. (2001), deterioration of GBS during postpartum follow-up were reported, too [28, 29]. MG and GBS both have fatal neonatal and maternal complications; hence, multidisciplinary care is required to prevent any serious complications. Brain tumors are rare during pregnancy, but hormonal changes and fluid retention in pregnancy might enlarge the tumor [30, 31]. Two cases of brain tumors were observed in the MCMC registration system. Expect gestational diabetes in one patient, no maternal complication was reported. The onset of symptoms was in the first and the second trimesters for each patient, which can be explained by the dramatic hormonal changes in these two trimesters.

## Conclusion

Neurological disorders are frequent during pregnancy. Migraine, seizure, benign intracranial hypertension and cerebral venous and sinus thrombosis are the most common neurological disorders in this period. The investigation, diagnosis, and management of neurological disorders during pregnancy is particularly challenging and requires multidisciplinary team, including neurologist, obstetrician and gynecologist, neurosurgeon, and pharmacologist.

## Acknowledgment

This study was financially supported by Shiraz University of Medical Sciences (grant number: 1396-01-67-16182). The authors wish to thank Mr. H. Argasi at the Research Consultation Center (RCC) of Shiraz University of Medical Sciences for his invaluable assistance in editing this manuscript.

## Conflict of Interest

The authors declare no conflict of interest in this study.

**Table 1:** Characteristics, Neurological Profile and Pregnancy Outcome of Patients with Neurological Condition.

**Parameters**	**Total**	**Parameters**	**Total**
**Age (Mean ±SD)**	29.32	GBS	1 (0.3%)
**Obstetric Profile (Mean ±SD)**		MG	4 (1.2%)
Gravidity	2.25	Eclampsia	16 (4.8%)
Parity	1.71	Other convolution	18 (5.4%)
MRS	0.2	**Onset of neurological symptoms**	
**Gestational diabetes, n (%)**		Before pregnancy	78 (23.5%)
Yes	30 (9%)	First trimester	43 (13%)
No	242 (72.9%)	Second trimester	42 (12.7%)
Unavailable	60 (18.1%)	Third trimester	62 (18.6%)
**Gestational hypertension, n (%)**		Pere-partum	5 (1.5%)
Yes	64 (19.3%)	Post-partum	76 (22.9%)
No	208 (62.6 %)	Unavailable	26 (7.8%)
Unavailable	60 (18.1%)	**Pregnancy outcome**	
**Neurological Profile, n (%)**		**Delivery mode**	
**History of neurological disorders**		NVD	102 (30.7%)
Yes	59 (17.8%)	C/S	203 (61.1%)
No	245 (73.8%)	Abortion	18 (5.4%)
Unavailable	28 (8.4%)	Unavailable	9 (2.7%)
**Neurological conditions**		**Fetus**	
Headache	179 (53.9%)	Abortion	18 (5.4%)
Epilepsy	57 (17.2%)	Stillbirth	6 (1.8%)
CVST	29 (8.7%)	Preterm	27 (8.1%)
Stroke	3 (0.9%)	Term	262 (78.9%)
ICH	8 (2.4%)	Congenital disorders	2 (0.6%)
MS	15 (4.5%)	Unavailable	17 (5.1%)
Brain tumor	2 (0.6%)	**Maternal mortality**	2 (0.6%)

**MRS:** Modifying ranking scale**; CVST:** Cerebral venous sinus thrombosis**; ICH:** Intracranial hemorrhage**; MS:** Multiple sclerosis**; GBS:** Guillen-barre syndrome**; MG:** Myasthenia gravis**; NVD:** Normal vaginal delivery**; C/S:** Cesarean section

**Table 2:** Characteristics, Neurological Profile and Pregnancy Outcome of Patients with Each Neurological Condition

**Parameters**	**Headache**	**Epilepsy**	**Seizure**	**Stroke**	**CVST**	**ICH**	**MS**	**Brain Tumor**	**GBS**	**MG**	**Eclampsia**
**Age**	30	28.6	27.77	29.3	29	27.75	29.2	34	31	26.2	27.25
**Obstetric profile**	
Gravidity	2.4	2.2	2.15	1.33	1.82	2	2.53	4	1	1	1.87
Parity	1.84	1.75	1.44	1.33	1.32	1.57	1.6	2.5	1	1	1.47
**Gestational diabetes** Yes No Unavailable	18 (10.1%) 131(73.1%) 30(16.8%)	5(8.2%) 41(67.2%) 15(24.6%)	2(14.3%) 7(50%) 9(35.7%)	0(0%) 3(100%) 0(0%)	1(3.4%) 19(69%) 8(27.6%)	2(25%) 5(62.5%) 1(12.5%)	0(0%) 14(93.7%) 1(6.7%)	1(50%) 1(50%) 0(0%)	0(0%) 1(100%) 0(0%)	0(0%) 1(100%) 0%	1(6.3%) 15(93.8%) 0(0%)
**Gestational hypertension** Yes No Unavailable	35(19.5%) 114(63.7%) 30(16.8%)	7(11.4%) 39(64%) 15(24.6%)	1(7.1%) 8(57.1%) 5(35.7%)	1(33.3%) 2(66.7%) 0(0%)	1(3.4%) 19(69%) 9(27.6%)	3(37.5%) 4(50%) 1(12.5%)	0(0%) 14(93%) 1(6.7%)	0(0%) 2(100%) 0(0%)	0(0%) 1(100%) 0(0%)	0(0%) 1(100%) 0(0%)	16(100%) 0(0%) 0(0%)
**Neurological profile**
**Previous neurological disorders** Yes No Unavailable	9(5%) 149(83.3%) 21(11.7%)	48(84.2%) 9(15.8%) -	1(7.1%) 4(71.4%) 3(21.4%)	0(0%) 3(100%) 0(0%)	1(3.4%) 27(93.2%) 1(3.4%)	0(0%) 8(100%) 0(0%)	14(93.4%) 1(6.7%) 0(0%)	1(50%) 1(50%) 0(0%)	0(0%) 1(100%) 0(0%)	4(100%) 0(0%) 0(0%)	0(0%) 16(100%*) 0(0%)
**Pregnancy outcome**
**Delivery mode** NVD C/S Abortion unavailable	58(32.4%) 106(59.2%) 9(5%) 6(3.4%)	55(43.9%) 27(47.4%) 4(7%) 1(1.7%)	3(16.7%) 14(77.7%() 0(0%) 1(5.6%)	0(0%) 3(100%) 0(0%) 0(0%)	7(24.1%) 21(72.4%) 0(0%) 1(3.4%)	1(12.5%) 16(75%) 1(12.5%) 0(0%)	4(26.7%) 8(53.3%) 3(20%) 0(0%)	0(0%) 2(100%) 0(0%) 0(0%)	0(0%) 1(100%) 0(0%) 0(0%)	1(25%) 3(75%) 0(0%) 0(0%)	3(18.7%) 12(75%) 1(6.3%) 0(0%)
**Fetus** Abortion Stillbirth Preterm Congenital disorders Term Unavailable	9(5%) 1(0.6%) 14(7.8%) 1(0.6%) 142(83.2%) 5(2.8%)	4(7%) 1(1.8%) 5(8.7%) 0(0%) 44(77.2%) 3(5.3%)	0(0%) 0(0%) 2(11.1%) 0(0%) 12(66.7%) 6(33.3%)	0(0%) 0(0%) 0(0%) 0(0%) 3(100%) 0(0%)	0(0%) 3(10.3%) 1(3.4%) 1(3.4%) 2(72.4%) 3(10.3%)	1(12.5%) 0(0%) 1(12.5%) 0(0%) 5(62.5%) 1(12.5%)	3(20%) 0(0%) 0(0%) 0(0%) 12(80%) 0(0%)	0(0%) 0(0%) 1(50%) 0(0%) 1(50%) 0(0%)	0(0%) 0(0%) 0(0%) 0(0%) 1(100%) 0(0%)	0(0%) 0(0%) 0(0%) 0(0%) 4(100%) 0(0%)	1(6.3%) 1(6.3%) 3(18.8%) 0(0%) 10(62.5%) 1(6.3%)
**Maternal mortality**	0(0%)	0(0%)	0(0%)	1(33.3%)	0(0%)	1(12.5%)	0(0%)	0(0%)	0(0%)	0(0%)	0(0%)
**MRS (±SD)**	1.02(±0.16)	1.03(±0.18)	1.00(±0.00)	3.16(±2.31)	1.03(±0.19)	1.00(±0.00)	2.06(±0.70)	1.00(±0.00)	4.00(±0.00)	1.50(±0.57)	1.18(±0.75)

**MRS:** Modifying ranking scale; **CVST:** Cerebral venous sinus thrombosis; **ICH:** Intracranial hemorrhage; **MS:** Multiple sclerosis; **GBS:** Guillen-barre syndrome; **MG:** Myasthenia gravis; **NVD:** Normal vaginal delivery;** C/S:** Cesarean section

**Table 3:** The Onset of Neurological Symptoms Separately Base on Duration of Pregnancy and Postpartum. Data Are Presented as n (%)

**Onset of neurological symptoms**	**Headache**	**Epilepsy**	**Seizure**	**Stroke**	**CVST**	**ICH**	**MS**	**Brain tumor**	**GBS**	**MG**	**Eclampsia**
**Before pregnancy**	9(5%)	48(84.2%)	2(11.1%)	0(0%)	1(3.4%)	0(0%)	14(93.3%)	0(0%)	0(0%)	4(100%)	0(0%)
**First trimester**	29(16.2%)	8(14%)	2(11.1%)	0(0%)	3(10.3%)	0(0%)	1(6.7%)	1(50%)	0(0%)	0(0%)	0(0%)
**Second trimester**	30(16.8%)	1(1.8%)	4(22.2%)	0(0%)	3(10.3%)	1(12.5%)	0(0%)	0(0%)	0(0%)	0(0%)	2(12.5%)
**Third trimester**	42(23.5%)	0(0%)	2(11.1%)	1(33.3%)	7(24.1%)	2(25%)	0(0%)	1(50%)	1(100%)	0(0%)	6(37.5%)
**Pere-partum**	1(0.6%)	0(0%)	2(11.1%)	0(0%)	1(3.4%)	1(12.5%)	0(0%)	0(0%)	0(0%)	0(0%)	0(0%)
**Post-partum**	47(26.3%)	0(0%)	3(16.7%)	2(66.7%)	13(44.8%)	4(58%)	0(0%)	0(0%)	0(0%)	0(0%)	7(43.7%)
**Unavailable**	21(11.6%)	0(0%)	3(16.7%)	0(0%)	1(3.4%)	0(0%)	0(0%)	0(0%)	0(0%)	0(0%)	1(6.3%)

MRS: Modifying ranking scale; CVST: Cerebral venous sinus thrombosis; ICH: Intracranial hemorrhage; MS: Multiple sclerosis; GBS: Guillen-barre syndrome; MG: Myasthenia gravis; NVD: Normal vaginal delivery; C/S: Cesarean section
